# Robustness in biomolecular simulations: Addressing challenges in data generation, analysis, and curation

**DOI:** 10.1016/j.xcrp.2025.102566

**Published:** 2025-04-30

**Authors:** Anne M. Brown, Justin A. Lemkul

**Affiliations:** 1 University Libraries, Virginia Tech, Blacksburg, VA 24061, USA; 2 Department of Biochemistry, Virginia Tech, Blacksburg, VA 24061, USA; 3 Center for Drug Discovery, Virginia Tech, Blacksburg, VA 24061, USA

## Abstract

Computational simulations of biomolecules provide a wealth of information about the thermodynamic landscape of biologically important systems, kinetics of important cellular processes, and the biophysical basis of life. Despite the ubiquity of molecular simulations in biophysical literature, major challenges persist for new practitioners entering the field, and even for experienced computational scientists, in maintaining and distributing their simulation outcomes. Here, we summarize critical obstacles encountered when performing biomolecular simulations and provide best practices for performing simulations that are robust, reproducible, and hypothesis-driven. We also discuss practices that promote improved reproducibility and accessibility using reliable tools and databases.

## INTRODUCTION

The field of biomolecular simulation lies at the intersection of biology, chemistry, physics, and computer science. As such, practitioners must develop skills ranging from quantum and statistical mechanics to programming and interaction with high-performance computing environments. Given the availability of numerous tutorials and resources to facilitate the preparation of these simulations,^1–6^ it is increasingly straightforward to execute the simulations. However, it is not as straightforward to meaningfully and carefully develop biomolecular simulation systems that are methodologically rigorous and answer the biological questions posed. Despite the reduced barriers to performing biomolecular simulations, numerous challenges remain in the proper collection, analysis, reporting, and curation of the associated data. Each of these requires expertise in domain-specific concepts. Our goals are complementary to recent reviews on simulation best practices,^7^ which provide foundational guidance on methodological rigor in biomolecular simulations.

**Figure 1.**
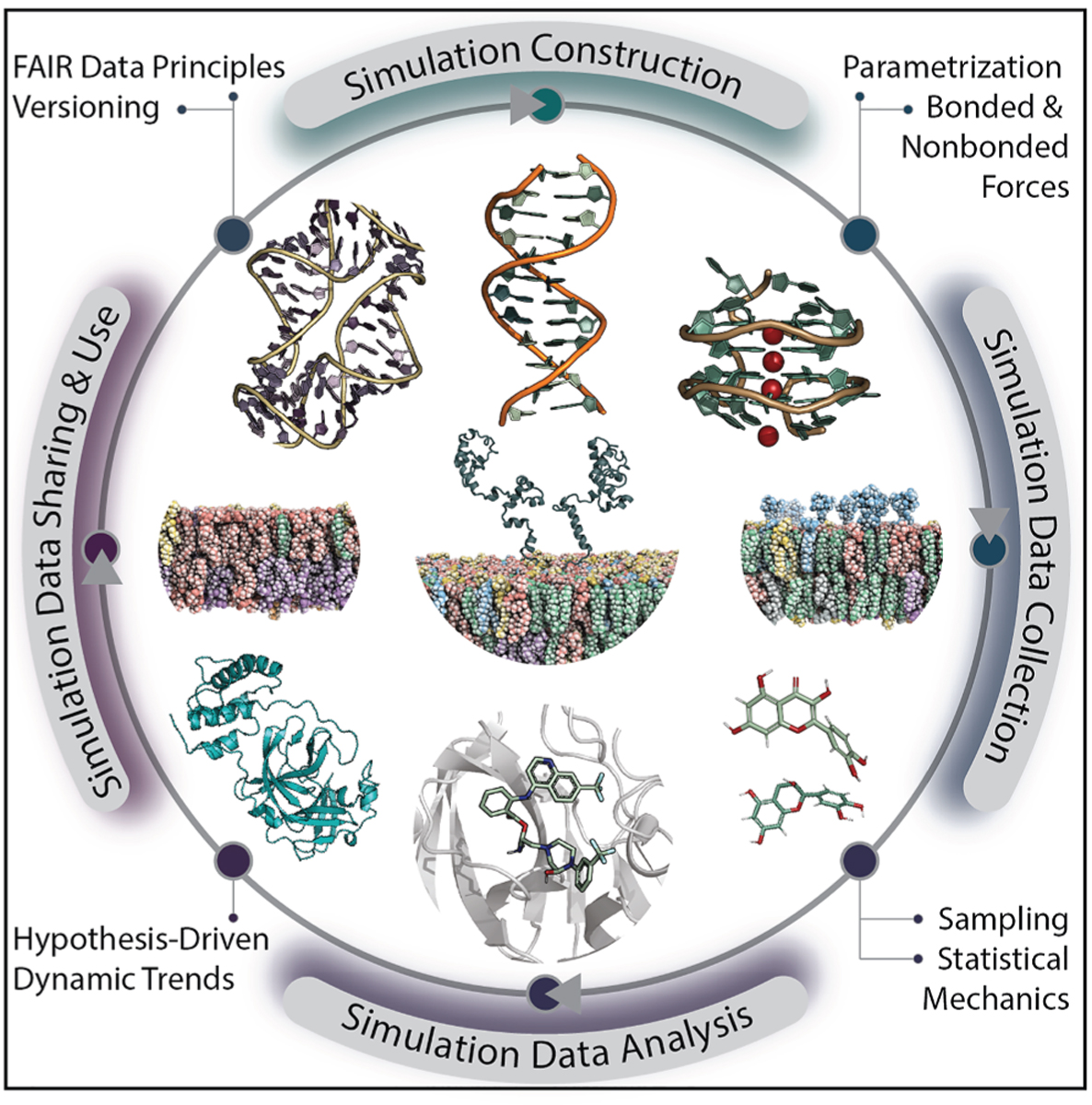
Overview of the biomolecular simulation process and best-practice considerations for each stage Biomolecular simulation workflows are inherently interrelated and iterative, from system construction and parametrization to analysis and dissemination, with a strong emphasis on hypothesis-driven scientific inquiry and attention to detail to ensure simulation accuracy and reliability. Here, we illustrate different examples of biomolecules in a visual representation, which is not reflective of actual simulation conditions.

In this perspective, we address some of the most critical challenges that arise throughout the process of preparing and reporting on outcomes of atomistic molecular dynamics (MD) simulations on biomolecules, all of which are ultimately interrelated (Figure 1). Here, our focus is on simulations driven by empirical force fields, as many of the challenges are similar to those of other computational research domains.

## CHALLENGES IN SIMULATION CONSTRUCTION AND DATA GENERATION

Some of the most crucial challenges in biomolecular simulations arise during the preparation of the system being studied. At this stage of the research project, these challenges will be largely associated with understanding how force fields are derived and how MD simulation algorithms are employed to ensure rigorous adherence to the principles of statistical mechanics.

### Integrity of the physical model is essential

The predictive power of any MD simulation depends on the validity of the model physics being employed. Most atomistic MD simulations make use of empirical force fields to compute energies and forces that propagate dynamics. As such, errors in the force field impact the simulation outcomes. In applying force fields for MD simulations, there are several important factors that users must keep in mind.

Force fields are self-consistent entities. That is, each has an established method for parameter development, often targeting quantum mechanical (QM) and empirical data.^8^ Moreover, these force fields are developed with specific algorithms used to compute bonded and nonbonded forces. Lastly, error cancellation may be important in the context of properly balancing intermolecular interactions. Ultimately, there are several aspects that users control when running a simulation that can undermine the validity of the chosen force field, perhaps unknowingly.

In this regard, there are several challenges that emerge. The first is in choosing appropriate methods for computing bonded and nonbonded forces. In the context of bonded interactions, it is important to appropriately assign constraints. Many algorithms have been developed to constrain bond lengths.^9–11^ A choice must be made in terms of which bonds are to be constrained. For most force fields, the constrained bonds are those involving hydrogen atoms, which have the fastest vibrational frequencies. Some force fields assume that all bonds are kept rigid. Misapplication of constraints can imbalance the force-field model in subtle ways. If bonds are kept rigid when they are expected to fluctuate, then the parametrization of associated bonded terms (e.g., angles and dihedrals) may be compromised and introduce small but accumulating errors, affecting conformational sampling.

The user must also choose the appropriate settings for nonbonded calculations, specifically cutoffs. While these values are freely tunable in any MD simulation software, the values employed for truncation of Lennard-Jones (LJ) and electrostatic interactions are often fixed properties of the system that yield an appropriate balance between the contributions of these forces to give an accurate intermolecular interaction energy. The particle mesh Ewald (PME) method^12^ largely removes the requirement for strict adherence to a short-range electrostatic cutoff, but the treatment of LJ terms may still be very sensitive to the choice of cutoff.^13^ The introduction of PME methods for computing LJ forces^14,15^ is an important development that will likely aid in resolving this issue. New users need to be aware that shortening cutoffs will speed up the simulation but will also almost certainly lead to a fast simulation with unreliable results. Moreover, force fields may need to be recalibrated to use the LJ-PME convention^16^; therefore, it cannot be assumed that existing force fields will be immediately compatible with this algorithm, and validation should always be performed.^17^

The second challenge is related to force-field parametrization itself. If a molecule of interest (modified biological residue, substrate, or other ligand, etc.) does not exist as a predefined entity in the force field, then the user must develop parameters for it. Doing so often requires knowledge of QM calculations, parameter fitting approaches, and validation against empirical data. Given the intense nature of these tasks, automated methods have been developed to assist users in parametrizing species of interest.^18–20^ For users seeking a balance between automated and manual parameterization, the force Field Toolkit plugin for VMD^21^ provides a guided interface to generate and refine parameters. Despite the availability of such tools, a major challenge remains in validating the resulting topology. Many users do not scrutinize the suitability of the topology and assigned parameters; instead, they rely on a “black box” acceptance of the automated methods. Where scoring functions exist to indicate topological quality, these should be used to the greatest extent possible. It should be understood that parametrizing a new species is a major undertaking that requires deep theoretical and practical knowledge rather than a trivial task that can be achieved in little time.

As an example, the CGenFF program produces parameter “penalty” values, which indicate the program’s confidence that the assignment of the new parameter is robust. The score is not inherently an indicator of quality; rather, it is only the apparent reliability of applying an existing parameter to a new set of atom types. High penalties mean there is a large chemical difference between some atom types, possibly necessitating modification. The user should then understand how to validate each parameter individually, e.g., through the calculation of either dipole moments and water interactions (in the case of charge penalties) or vibrational motions or potential energy scans (for internal terms). In reporting on simulations with newly developed parameters, the topology and parameter files should always be provided publicly in a machine-readable format, and the efforts to validate and refine parameters, if necessary, should be reported in the published article.

### Sampling, sampling, sampling

Another major challenge in ensuring the reliability of MD simulation data is the adequacy of the sampling. In an ideal case, each MD simulation trajectory would be ergodic; that is, the time averages of the properties of interest equal the ensemble averages. Achieving such a condition requires essentially infinite simulation time, which is obviously impossible. Therefore, MD simulation practitioners must achieve sufficient sampling of the energy surface of interest. A single simulation trajectory has a tendency to get “stuck” in a local energy minimum, given that larger conformational changes are unlikely on the typical ns-μs timescale within the limits of thermal fluctuations.

One approach to overcoming this challenge is to perform replicate simulations.^22^ Just as one would conduct an experiment several times to prove its robustness, so too should MD simulations be repeated.^23^ These replicates can be initiated from different random velocities at the outset of the protocol or from different starting configurations of the system. By doing so, each simulation begins at a slightly different point in phase space and, therefore, may sample somewhat different features of the energy landscape and, thus, states within the statistical mechanical ensemble, thereby better approximating ergodicity. Other possible solutions involve using biased simulation methods, such that slow degrees of freedom are sampled more quickly, and more states are visited. A variety of such approaches exist, including metadynamics,^24^ Gaussian-accelerated MD,^25^ and weighted ensemble methods.^26^ A full description of these techniques is beyond the scope of this perspective.

Further, the simulation timescale itself is a key consideration. It is tempting to directly follow the prescribed methodology, from either previously published studies or tutorials, and use it as precedent for determining an appropriate trajectory length. A common limitation of tutorials is that they prioritize speed over realism, often employing short simulations for demonstration purposes. Readers should be cautious and adapt such protocols to their scientific needs. A key consideration here is the intrinsic dynamics of the system that are of interest. Some biomolecular processes are fast and, therefore, may be easily observed multiple times in a single trajectory or over several replicates. Other dynamics are much slower and require extremely long simulations, many replicates, or biasing forces to observe. Investigators should carefully consider these aspects when planning the length of their simulations; there is no “default” selection for a simulation length because this property is inherently linked to the dynamics in the system. In addition, it is critical to assess the convergence of the simulation; that is, analyzing quantities of interest to ensure that they are not systematically varying with time. If adequate convergence has not been achieved, then the simulation outcomes lack robustness. Other details and considerations regarding structural convergence and sampling assessment have been reviewed elsewhere.^27^

Lastly, in assessing the states visited during the simulation, it is important not to “cherry-pick” snapshots to simply show preferred states. In many respects, these manually identified states may not be truly representative of the behaviors observed over the course of the trajectory. MD simulations should be rigorously analyzed to determine the most representative sub-states that have been visited using methods like root-mean-squared deviation (RMSD) clustering,^28,29^ t-distributed stochastic neighbor embedding (t-SNE),^30^ dimensionality reduction techniques,^31^ *k*-means clustering,^32^ or Bayesian methods,^33^ for example. Additionally, to aid in dimensionality reduction and trajectory interpretation of these representative structures, we recommend also considering physically meaningful methods such as principal-component analysis (PCA),^34^ nonlinear techniques like uniform manifold approximation and projection (UMAP),^35^ or Sketch Map,^36^ which better preserve inter-cluster relationships without impairing physical interpretations.

### Beware default settings

The predictive capabilities of MD simulations rely primarily on their connection to statistical mechanics. That is, the probabilities of observing certain states in an MD-generated trajectory define the thermodynamics in accordance with partition functions. Therefore, the energy surface sampled during the simulation must rely on appropriate model physics. The force field itself is a component of this requirement but so too is the set of algorithms employed to perform the simulation. These algorithms include the treatment of nonbonded forces, thermostats and barostats, time steps, and the use of constraints, among other features.

Many programs have a set of default settings that are enabled if the user does not make any other selection. These default settings are not necessarily physically valid; instead, they are intended to produce syntactically complete input files. A prominent example of this practice is the weak-coupling thermostat method of Berendsen et al.,^37^ a method that was routinely used for decades. Though capable of quickly scaling velocities to reach the desired temperature, it yields an ensemble of kinetic energies that is inconsistent with the expected canonical ensemble.^38^ As such, users should not rely on such algorithms, even if they remain available in the software and are often presented as a default choice.

A related issue is the reliance on tutorials to provide universally applicable methods for carrying out simulations. As noted above, input settings such as nonbonded cutoffs, use of constraints, etc., are often tied directly to the force field chosen for the simulation, and the length of the simulation inherently depends on the timescale of the relevant dynamics. Therefore, there is no single methodology for performing an MD simulation. As such, tutorials, while useful for training purposes, do not constitute “universal” methods that can be applied without changes. These exercises are often designed for a specific didactic purpose with specific considerations for suitability that new users may not fully appreciate.

## CHALLENGES IN SIMULATION DATA ANALYSIS

### Hypothesis-driven MD simulations and analysis are essential

When performing simulations of biomolecules, one should initiate the work with a research question in mind. Doing so will ensure that the system construction, force-field selection, and other essential parameters are appropriate for the biomolecule and environment that the research question seeks to answer. Utilizing a hypothesis-driven approach to MD simulations ensures that the data generated align with the specific scientific questions being addressed and utilize the most accurate system construction and parameters. Without a clear hypothesis, simulations risk producing data that are unfocused, irrelevant, or, worse, incorrect. A well-defined research question should guide not only the simulation setup and experimental design but also the subsequent analysis. In fact, biological relevance should be considered at the beginning of simulation design. Defining the key biological questions in advance allows simulations to be structured to contain the necessary controls and consider environmental influences such as pH, protonation states, salt concentration, etc. This approach shifts the focus from simply running standard protocols or procedures from a tutorial to actively probing biological mechanisms, answering questions that matter, and advancing the field of understanding at the atomistic level. For instance, instead of just assessing “protein stability” through RMSD, one could ask, “What conformational states are associated with ligand binding, and are they different from those of the unbound protein structure?” Such targeted questions help focus the analysis on novel insights, preventing over-reliance on default metrics.

### Beyond RMSD and RMSF: Employing advanced analysis techniques

While analyses like RMSD and root-mean-squared fluctuation (RMSF) are commonly used to assess structural deviations in a structure over time, they are often insufficient for providing a comprehensive understanding of molecular behavior. These metrics, though helpful, are basic and can miss important structural transitions or dynamic events. For example, RMSD is frequently used as a proxy for conformational stability, reflecting average atomic displacements over time as compared to the starting structure. However, this is problematic when defining conformational stability or states sampled, as it is not a quantitative definition of structural stability based on thermodynamics. More advanced techniques like clustering,^29,30,33,39^ PCA,^34^ time-lagged independent component analysis (tICA),^40,41^ or Markov state models (MSMs)^42^ should be used to capture and characterize the full range of biomolecular conformational states. Machine-learning-based clustering techniques, such as t-SNE,^30^ enable a more refined exploration of complex biomolecular conformation states, particularly in identifying rare or transient conformations. This deeper analysis moves beyond static snapshots, which are inadequate in conferring confidence of a conformational state sampled over time, to reveal dynamic trends that better reflect the true behavior of the biomolecular system. Other dimensionality techniques, like the energy landscape visualization method (ELViM),^31^ may also be useful in this regard. A more robust assessment of individual states can help drive other analyses that are specifically related to the biomolecule of interest, such as hydrogen bonding, secondary structure evolution, and rearrangements in internal geometry that lead to conformational change. Indeed, as each simulation project poses unique questions, custom analysis is often necessary. Utilities such as the TCL scripting interface in VMD,^43^ MDAnalysis,^44^ MDTraj,^45^ and LOOS^46,47^ support flexible, programmatic data interpretation.

### Clear figures for accessible and accurate data representation

It is essential to have accessible and legible figures to effectively communicate the results of MD simulations. Many researchers default to software-generated plots that might mimic tutorials or achieve bare-minimum standards without considering clarity or audience comprehension. Instead, figures should be tailored to highlight key findings, avoiding overly complicated or overly dense visualizations. For example, adjusting color schemes, adding clear annotations, and ensuring that axes are legible and labeled properly can transform a confusing plot into a clear description of the data being presented. Doing so is especially critical when presenting multi-dimensional data or advanced analyses like free energy landscapes, which require thoughtful presentation to convey the complexity of the results. Tools like Matplotlib^48^ or PyMOL^49^ offer the flexibility to create high-quality visualizations, and researchers should take advantage of their customizable features. Visualizations can further be improved by selecting color schemes that are colorblind friendly^50^ and by using detailed labels and legends to make them comprehensible. Additionally, using file formats such as SVG for vector graphics or high-resolution PNG ensures that figures retain their quality in publications or presentations.

### Contextualizing data to explain biological outcomes

Many MD simulation tutorials focus on basic analyses like RMSD or hydrogen-bonding patterns. However, these tutorials should serve as a starting point, not the final step. Researchers must go beyond these basics by tailoring their analyses to the unique features of their system. For example, using metadynamics or free energy perturbation methods to explore energy barriers or binding affinities can reveal deeper insights to more appropriately address the research question. Asking specific research questions and developing customized analysis workflows ensures that the simulations produce high-quality, actionable data rather than generic metrics. Additionally, interpreting MD simulation data requires more than presenting raw results—it demands contextualization within the broader biological system. It is not enough to state that a protein has a stable RMSD; researchers must interpret how this “stability,” which is really a thermodynamic concept that cannot be derived from RMSDs, using several metrics of analysis, affects biological function or interaction. Analyses should support conclusions about biomolecular conformational changes or molecular interactions. For example, instead of merely noting that a protein-ligand complex is stable, the analysis should explore the specific interactions or conformational changes driving that interaction. If hydrogen bonds are essential for ligand binding to a protein structure, do not simply list the residues involved in the hydrogen bonds. Rather, discuss the importance of a biomolecular interaction type, the duration, and relevance to the biological function. This practice ensures that the data interpretation is meaningful and scientifically relevant, providing actionable insights into the molecular system being studied.

## CHALLENGES IN DATA CURATION AND AVAILABILITY

OK, so you performed a simulation of a biomolecule, but how do people reproduce it or use it? The FAIR data principles—findability, accessibility, interoperability, and reusability—are a set of guidelines designed to improve the management and stewardship of research data. In the context of MD simulations, acknowledging and adhering to these principles ensures that data can be easily found, accessed, integrated, and reused by other researchers. These issues have recently been discussed by Caparotta and Perez^51^ and a consortium of leaders in the simulation field.^52^ Overall, providing standardized protocols for data preprocessing and analysis allows others to replicate results more easily. In addition, developing application programmable interfaces (APIs) or tools for automatic data extraction, simulation, and analysis can enhance accessibility, albeit at the cost of accuracy and appropriate contextualization of the data created. Encouraging community-driven contributions and feedback through platforms that support open-source collaboration can further amplify the value of the shared data, fostering innovation and iterative improvements in simulation methodologies.

### Reuse of input files and ensuring simulation reproducibility

To maintain FAIR data principles and be good stewards of generating high-quality, accurate MD simulation, all input files, parameter files, and key structural snapshots from the simulation should be shared. Sharing partial datasets in unusable file formats, such as only providing ligand topologies in a PDF, limits others’ ability to reproduce the work and ensure the accuracy of the simulation performed. To adhere to the FAIR data principles, researchers should provide complete input files in usable (and machine-readable) formats, such as plain-text input files, initial coordinates, and topologies for programs such as AMBER,^53^ CHARMM,^54^ GROMACS,^55^ NAMD,^56^ OpenMM,^57^ etc. These files should include full system topology, force-field parameters, and configuration settings. Doing so helps to address challenges in storing metadata associated with the simulations themselves, including algorithms used and force-field terms. Repositories such as GitHub, Zenodo, MDDB,^58^ and MDRepo^59^ offer structured platforms for sharing these data. Tools such as Martignac^60^ are a model for how to “work backward” from existing simulation data to determine how simulation systems were prepared, and additional effort should be placed on developing such tools. Proactive planning in terms of data sharing will facilitate reuse and reproducibility efforts over time.

### Sharing data from MD simulations

Fragmented storage and lack of proper repositories have long been challenges in MD simulations, but emerging platforms like MDDB, MDRepo, Zenodo, and GitHub offer robust solutions for sharing data. These platforms provide the necessary infrastructure for large datasets, support domain-specific metadata, and facilitate efficient data sharing. When storing large simulation files (e.g., XTC, TRR, or DCD), researchers should use downsampling and removal of solvent (if appropriate) to optimize storage space and file transfer rates. Plain-text files may benefit from file compression (e.g., ZIP, TAR, or GZ). Importantly, these repositories offer version control capabilities that allow researchers to track changes to their simulation setups and input files. While sharing trajectories and full simulation files is a standard we should be working toward, at minimum, researchers with simulation data should provide them in more manageable, easy-to-share files. Such files include initial simulation structures, software-specific input files, and essential representative structures (e.g., from clustering) for improved data sharing and transparency. This feature is particularly crucial for maintaining data interoperability and ensuring that future research can easily integrate and build on these simulations.

Preserving MD simulation data over time is vital for ensuring long-term usability and reproducibility. The National Institutes of Health and National Science Foundation require that raw data (e.g., trajectory files, initial coordinates, topologies, and input files) be preserved for at least 3 years, which we suggest as a best practice. This requirement is especially important since these files are crucial for reanalysis and reproducing original conditions. After this period, cleaned and concatenated trajectory files should be archived for longer periods, as they take up less space and retain essential information about the simulation. For long-term storage, using compressed file formats helps maintain data integrity while optimizing space.

### Importance of versioning

Proper reporting of software and file versions is crucial for reproducibility. Versioning therefore relates to (1) the versions of all software used and (2) the specific revision of key files, such as force-field parameters and input files. Here, we suggest two key points in ensuring data reproducibility.

For software versioning, it is critical that all versions of the software used to generate or analyze simulation data are reported in the corresponding publication. Doing so allows other practitioners to use the same software to, ideally, produce the same findings. More specifically, as software development continues over time, features are changed and improved, and bugs are fixed. Both activities can lead to different results when trying to reproduce scientific work.

For archiving input files, force-field parameters, or other data, using a platform that allows for versioning is critical. Maintaining an open-access, online repository is a good practice, but keeping a record of any changes made over time is even more critical. Just as in the case with software versioning that allows for an understanding of potential impacts on simulation outcomes, revising input files for future use should be done only with corresponding documentation (e.g., via Git commits that explain the nature of the change) so future users can understand how inputs may have changed due to new features or enhancements or how force-field files change due to ongoing development. The use of a platform like Git also allows a user to go “back in time” to use a specific version that corresponds to a published work, particularly through the use of version tagging.

## CONCLUSIONS

Here, we have presented several common challenges faced by scientists who perform biomolecular simulations. We provide this perspective for the benefit of new users entering the field and for seasoned experts to consider how they can amplify the robustness and accessibility of their work to enhance reproducibility and usability. This list of suggestions is by no means exhaustive but should help alleviate many of the common problems encountered during the process of engaging in theoretical research. Several of these suggestions may also be useful in other computational domains beyond the force-field-based biomolecular simulations in which the authors specialize.

To summarize our perspective, we strongly encourage all practitioners of biomolecular simulations to keep the following key points in mind.

1. Physical integrity: force-field parametrization must be approached with great care to ensure the consistency of the model’s physics, and algorithmic settings must be chosen to ensure the validity of the integration during MD simulations.
2. Sampling: replicate simulations of sufficient length are essential and, even then, may be inadequate to fully answer the question(s) at hand. In that case, enhanced sampling methods should be employed. While enhanced sampling is outside the scope of this manuscript, we direct interested readers to a recent comprehensive reviews on this topic.^61^
3. Input settings: software defaults often reflect only syntactical correctness rather than physical correctness and must be scrutinized carefully.
4. Importance of hypotheses: simulations are experiments and should be approached with the same level of rigor as wet-bench work. Hypotheses should be tested and specific questions designed that can be addressed via simulations.
5. Analysis: moving beyond tutorial analysis methods is essential, especially in light of answering challenging scientific questions. There is no “recipe” for analysis; everything should be planned with the specific system in mind.
6. Figure clarity: adjustments to default color schemes should be considered, especially for colorblind readers, and clarity is paramount given the vast amount of data points produced by MD simulations.
7. Biological context: the relationship of results to biological properties is linked to the importance of clear hypotheses and performing appropriate analysis. Simulations are best employed to generate new hypotheses/predictions or to rationalize existing behaviors with unknown mechanisms. Discussing simulation results in the context of experimental literature is essential.
8. Input files and sharing: openness and reproducibility are key to the success of biomolecular simulations. Investigators should make use of convenient file-sharing platforms to provide raw input files and code to exactly reproduce published findings.
9. Versioning: specific versions of software, force fields, and any other auxiliary tools should be documented to ensure reproducibility.
